# Antimicrobial and food barrier properties of polyvinyl alcohol–lactic acid food packaging films

**DOI:** 10.1002/fsn3.4291

**Published:** 2024-06-21

**Authors:** E. S. Madivoli, J. Kisato, J. Gichuki, C. M. Wangui, P. K. Kimani, P. G. Kareru

**Affiliations:** ^1^ Department of Chemistry Jomo Kenyatta University of Agriculture and Technology Nairobi Kenya; ^2^ Department of Fashion and Design Kenyatta University Nairobi Kenya; ^3^ Pharmaceutical Chemistry Department Mount Kenya University Thika Kenya; ^4^ Department of Engineering, Graduate School of Engineering Gifu University Gifu Japan

**Keywords:** anthocyanin, food packaging, pH responsive

## Abstract

Microbial contamination and the need for sustainable food production are driving the shift toward biodegradable food packaging materials. There is an urgent need to develop smart food packaging materials that can prevent contamination and prolong the shelf life of meat. To achieve this, the physical–chemical characteristics of polyvinyl alcohol (PVA)‐based packaging films were enhanced through incorporation of lactic acid and anthocyanins to act as a pH indicator. The mechanical, hydrophilic, barrier, and antibacterial properties of the composite films were then evaluated to test the ability of the film to act as a packaging material. In addition, the surface morphology was studied by scanning electron microscopy (SEM), the functional groups by Fourier transform infrared (FTIR) spectroscopy, optical transparency using ultraviolet–visible (UV–vis) spectrophotometer, crystallinity by powder diffraction, and their thermal properties by thermal gravimetric analysis (TGA). The films had a swelling degree (SD) of 222.60 ± 21.19%, dry content (DC) of 70.56 ± 2.54%, moisture content (MC) of 29.44 ± 2%, ALRO moisture (AM) content of 41.85 ± 5.06, and total soluble matter (TSM) of 8.05 ± 1.05%. Moreover, incorporation of lactic acid enhanced the mechanical and the thermal properties of the films but it reduced their optical transparency. The water vapor permeability (WVP) was found to be 14.32 × 10^−3^ g^−1^ s^−1^ Pa^−1^ and it inhibited the growth of *Escherichia coli* (EC) (10.67 ± 0.58 cm), *Staphylococcus aureus* (SA) (10.50 ± 0.40 cm), *Pseudomonas aeruginosa* (PA) (10.33 ± 0.58 cm), and *Staphylococcus epidermidis* (11 ± 1 cm) but not *Bacillus subtilis* (BS). The film's hue changed from red to green over time when used as a packaging material for meat under ambient condition indicating a deterioration in freshness. In conclusion, the developed packaging film exhibited enhanced mechanical, antimicrobial, and hydrophilic properties and it can be used to store and relay information when stored meat begins to decompose through a visible color change of the films.

## Graphical abstract

Conventional food packaging materials lead to food contamination with microplastics. Biodegradable polymers are an alternative to these plastics. Polyvinyl acid/lactic acid films are a promising replacement for food packaging materials. Developed films have good mechanical properties and are able to detect meat decay.

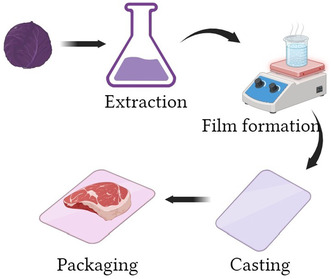

## 1 INTRODUCTION

The presence of microplastics from single‐use packaging material necessitates the need to develop biodegradable intelligent food packaging to curb environmental degradation, food waste associated with food decay, and to enhance food safety (Danopoulos et al., 2020; Eriksen et al., 2014). Even though there are increasing legislative measures in place to curb the use of single‐use plastics, little is being achieved with this approach. Nevertheless, plastics are increasingly being detected along the food chain as microplastics, in human blood and placenta with unknown health effects (Eriksen et al., 2014; Kumar et al., 2022; Leslie et al., 2022). To this end, it is becoming paramount that compostable and biodegradable packaging materials be developed to replace single‐use plastics, such as polyvinyl chloride (PVC) and polyethylene (PE), which are currently used in food packaging. To do so, agricultural residues and feedstocks, such as banana pseudo stems, pineapples, and rice husks, which are renewable, biodegradable, and compostable, are an excellent feedstock for the production of cellulose, lactic acid, carrageenan, and other polymers. These polymers are not only renewable in line with the principle of circular economy but they also enable us to satisfy several sustainable goals when they are utilized to produce bioplastics to replace petroleum‐based plastics. To this end, several authors have reported excellent findings on the properties of bioplastics that have been developed from seaweed (Singh et al., 2020; Yegappan et al., 2018), cellulose (Mishra et al., 2018; Otenda, Kareru, Madivoli, Salim, et al., 2022), chitosan (Afonso et al., 2019; Romanazzi et al., 2017), pectin (Prameela et al., 2018; Singh et al., 2020), lactic acid, and polylactic acid (Jamshidian et al., 2012; Prameela et al., 2018), among other biopolymers. These materials have been observed to have good mechanical properties, are biodegradable and compostable, and are biocompatible, hence they are currently being used as food packaging, feminine ware, biomedical applications, and in agriculture, among other industries (Otenda, Kareru, Madivoli, Maina, et al., 2022; Otenda, Kareru, Madivoli, Salim, et al., 2022; Saenjaiban et al., 2020; Souza & Fernando, 2016). Their water vapor permeability (WPV), oxygen barrier properties, ultraviolet (UV)‐blocking behavior, mechanical properties, biodegradability, biocompatibility, and thermal stability are crucial parameters that govern their final application (Saenjaiban et al., 2020; Souza & Fernando, 2016). For instance, the influence of plasticizer and cellulose nanofibrils (CNF) content on the physical, structural, and morphological properties of tomato mucilage biodegradable film has been reported (Ghadiri Alamdari et al., 2021). In this study, the authors observed that while the thermal stability of the resultant films increased due to the presence of glycerol and CNF, the water barrier properties and oxygen permeability were negatively affected by high glycerin content (Ghadiri Alamdari et al., 2021). To impart them antimicrobial properties, the composite films made from biopolymers have been infused with secondary metabolites isolated from several plants, metal/metal oxide nanoparticles, or even antimicrobial peptides, which make them resistant to microbial contamination (Cruz‐Gálvez et al., 2018; Otenda, Kareru, Madivoli, Salim, et al., 2022). Moreover, incorporation of stimuli‐responsive moieties such as anthocyanins makes them responsive to stimuli, such as pH, temperature, moisture, and biogenic amines like dimethyl amine and methyl amine. These biogenic amines are formed as a result of amino acid decarboxylation when meat is under storage, in which substrate‐specific enzymes break down proteins (Erkmen & Faruk Bozoglu, 2016; Miller et al., 2023). In this regard, pH‐responsive food packaging materials are a smart and innovative strategy for detecting, tracking, and protecting the quality of food, and it is an ultimate tool for consumer protection (Khoo et al., 2017; Luo et al., 2023; Roy & Rhim, 2021). Hence, this study sought to develop a pH‐responsive and antimicrobial composite film using polyvinyl alcohol (PVA)–lactic acid (LA) blends infused with secondary metabolites isolated from *Brassica oleracea* var. *capitata* for food packaging. The changes in functional groups upon esterification, optical properties, water vapor permeability, and crystallinity were studied using Fourier transform infrared (FTIR) spectrophotometer, ultraviolet–visible (UV–vis) spectrophotometer, and powder diffraction. The stimuli‐responsive behavior of the film against ammonia, methyl amine, trimethyl amine, and sodium hydroxide was evaluated by studying the colorimetric response of the films against these chemicals with pH values of 11, 12, 13, and 14, respectively.

## 2 MATERIALS AND METHODS

### 2.1 Extraction of anthocyanins from *B. oleracea*

Bioactive compounds identified in *B. oleracea* have been shown to possess antioxidant activity, contribute to human health, prevent oxidative process occurring during food storage, and change color at different pH values. In the case of food packaging, as pH is one of the factors that can be used in the evaluation of food spoilage, their incorporation into PVA–LA matrices imparts this ability to the resultant film. To achieve this, *B. oleracea* was first milled in a household blender and sieved using a 40‐mesh size sieve to obtain a dry powder that was stored in the dark in a Winchester bottle. Subsequently, methyl alcohol was added to the ground samples at the solid–liquid ratio of 1:20 (g:mL), macerated for 24 h, filtered, and the solvent removed by rotary evaporation (Madivoli et al., 2018; Ruto et al., 2018). The anthocyanin extract was then fractionated in hexane and ethyl acetate and subjected to gas chromatography–mass spectrometry (GC–MS) analysis for compound identification before incorporation of the methanolic extract into the composite films (Madivoli et al., 2018; Ruto et al., 2018). Gas chromatographic (GC) analysis was carried out on Agilent 5975 GC–MS operating in electron ionization (EI) mode at 70 eV. A capillary column of 30 m × 0.25 mm (internal diameter [id]) was prepared and helium gas with a flow rate of 1.2 mL min^−1^ was used as a carrier gas and oven temperature of 60°C. Various compounds were identified by their retention time and the National Institute of Standards and Technology (NIST) library search (Madivoli et al., 2018; Ruto et al., 2018).

### 2.2 Preparation of the PVA/LA composite film

**FIGURE 1:**
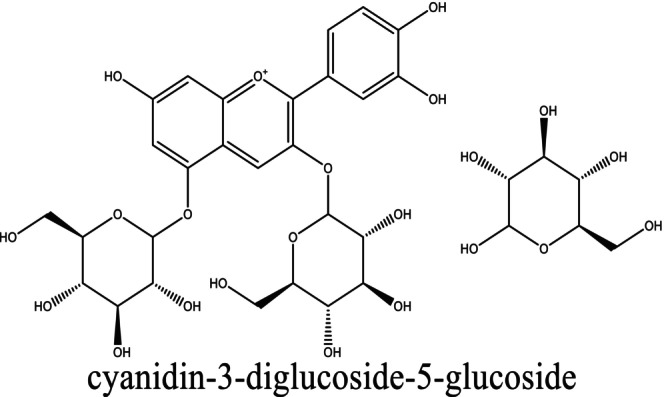
The major constituent of methanolic extracts of *Brassica oleracea* var. *capitata*.

**SCHEME 1:**
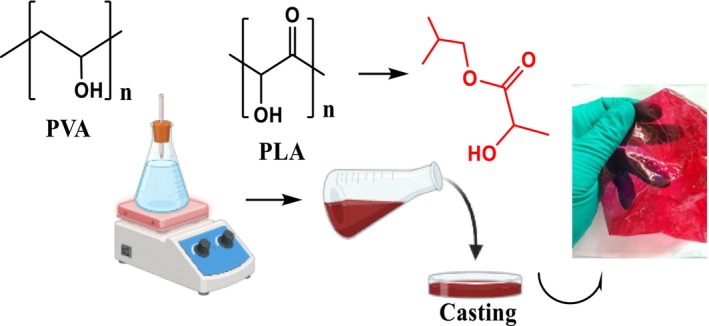
Preparation of pH‐responsive composite films infused with *Brassica oleracea* var. *capitata* extracts.

The composite film was prepared according to an optimized PVA–LA ratio, as reported in the literature where a higher concentration of LA reduced mechanical strengths of resultant films. In this case, 5 g of PVA and lactic acid was dissolved in deionized water at 50°C for 1.5 h to obtain a PVA/LA solution (1:1 m/m). To remove air bubbles, the viscous solution was ultrasonicated in an ultrasonic bath for 1 h, poured in polytetrafluoroethylene (PTFE) molds, and dried in an oven at 50°C to constant weight. On the other hand, films infused with anthocyanins (Figure 1, Scheme 1) were prepared in a similar manner but incorporating methanolic extracts of *Brassica oleracea* var. *capitata*.

### 2.3 Hydrophilic properties

The moisture content (MC), dry content (DC), ATRO moisture (AM), swelling degree (SD), and total soluble matter (TSM) were used to evaluate the hydrophilicity of the films using a three‐step gravimetric method. First, to determine the MC and DC, the film samples with a surface area of 6 cm^2^ were weighed (M_1_), dried at 100°C for 24 h, and weighed again (M_2_) (Janik et al., 2023). The MC and DC were calculated using Equations 1 and 2.

(1) $$\mathrm{MC}=\frac{M_{1}-M_{2}}{M_{1}}\times 100$$

(2) $$\mathrm{DC}=\frac{M_{2}}{M_{1}}\times 100$$

(3) $$\mathrm{AM}=\frac{M_{1}-M_{2}}{M_{2}}\times 100$$

The swelling degree (SD) was evaluated by placing the film samples in 30 mL of distilled water at room temperature for 24 h and weighing again (*M*_3_).

(4) $$\mathrm{SD}\;\left(\%\right)=\frac{M_{3}-M_{2}}{M_{2}}\times 100$$

In the final step, TSM was determined by drying the samples at 100°C for 24 h and weighed (*M*_4_). Measurements were repeated five times, and the average value was calculated. TSM values were calculated using the following formula (Janik et al., 2023):

(5) $$\mathrm{TSM}\;\left(\%\right)=\frac{M_{2}-M_{4}}{M_{2}}\times 100$$

### 2.4 Tensile strength measurement of the composite films

To evaluate the mechanical properties of the composite films, the rectangular film (10 × 60 mm) specimens were clipped onto two small metal brackets, which were then clamped to a slotted mass hanger where 5 g weights were added periodically until film breakage (American Society for Testing and Materials, 2018; Stevens & Poliks, 2003). After each addition, the films' elongation was recorded and the total weight at film breakage was also recorded. The tensile strength, strain, and stress of the films were then calculated using the following equations (American Society for Testing and Materials, 2018; Stevens & Poliks, 2003):

(6) $$\mathrm{ts}\;\left(\mathrm{Pa}\right)=\frac{W\;\left(\mathrm{kg}\right)\times \left(9.80\;N\;{\mathrm{kg}}^{-1}\right)}{A\;\left(10^{-4}\;m^{2}{\mathrm{cm}}^{-2}\right)}$$

(7) $$\text{strain}\;\left(\varepsilon \right)=\frac{L-L_{0}}{L_{0}}$$

(8) $$\text{Stress}\;\left(\sigma \right)\;\left({\mathrm{Nm}}^{-2}\right)=\frac{\text{Load}}{\text{Cross sectional area}}$$

where *L* = length after applied load and *L*_0_ = initial length.

### 2.5 Water vapor permeability (WVP)

To evaluate the WVP of the films, the film sample was sealed over a circular opening 0.003 m^2^ in a permeation cell that was stored at 25°C in a desiccator with a water vapor partial pressure of 3106.51 Pa. To evaluate the water vapor partial pressure to a 98% relative humidity (RH) gradient across the film, anhydrous calcium chloride (2% RH) was placed inside the cell and distilled water (100% RH) was used in the desiccator. The test cell was periodically weighted after steady‐state conditions were reached. The WVP (g m^−1^ s^−1^ Pa^−1^) was calculated as follows:

(9) $$\mathrm{WVP}=\frac{∆w\times x}{A\times ∆t\times ∆P}$$

where ∆*w* is the test cells' weight gain, *x* is the film thickness, and *A* is the exposed area (0.003 m^2^) during duration ∆*t* under a water vapor partial pressure ∆*P*.

### 2.6 Detection of biogenic amines

**FIGURE 2:**
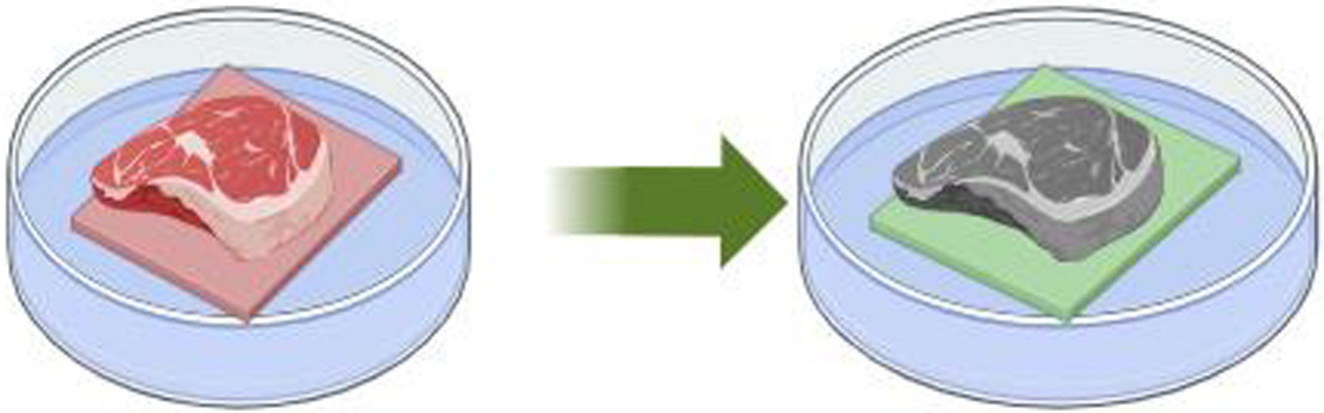
Detection of biogenic amines in meat under storage.

To test the applicability of the composite films as an indicator of spoiled food, 10 × 10 cm films were used to wrap beef, which were then stored under ambient conditions (Figure 2).

A change in the color of packaging films upon exposure to biogenic amines can be evaluated by a colorimeter or a colorimetric spectrophotometer. However, the most popular hardware‐oriented space is the standard RGB (red, green, blue), because it is least affected by a film's physical characteristics, such as thickness, microstructure, and surface concentration. Photographic images of the films used to wrap beef were taken periodically for 5 days (Acevedo et al., 2012). The color change in the thin strips was evaluated using digital images of the films before and after exposure to the beef. The color transition of the films was monitored by taking photographs with a smartphone camera (Redmi Note C) located 30 cm away in manual mode. To calculate the red chromatic shifts (RCS), the red–green–blue (RGB) values of the films were measured using ImageJ 1.48v software and Java‐based open‐source software developed at the National Institutes of Health (NIH) (Acevedo et al., 2012). The red intensity was then expressed in terms of red chromaticity (*r*) as follows:

(10) $$r=\frac{R}{R+G+B}$$

where *R* (red), *G* (green), and *B* (blue) are the values of three primary colors. The RCS (%) of composite films was calculated by comparing the red chromaticity of the composite film (*r*_sol_) to those of the blue (*r*_0_) and red (*r*_max_) phases of the films after contact with biogenic amines:

(11) $$\mathrm{RCS}\;\left(\%\right)=\frac{r_{\mathrm{sol}}-r_{0}}{r_{\max}-r_{0}}\times 100$$

here, *r*_0_ was measured from the wine red films immediately after polymerization and *r*_max_ was measured from green films after wrapping beef for 5 days (Jang et al., 2023).

### 2.7 Characterization of PVA–LA composite films

To determine changes in the functional groups present in the films, infrared (IR) spectra of the composite films were acquired using a Bruker Tensor II FT‐IR spectrophotometer (Bruker, Ettlingen, Germany) in the frequency range of 4000–400 cm^−1^. A Bruker D8 Advance Diffractometer (Bruker, Ettlingen, Germany) operating a copper tube under a voltage and current of 40 kV and 40 mA was used to acquire X‐ray powder diffractograms of the films (Madivoli, Kareru, Gachanja, et al., 2022; Madivoli, Kareru, Gichuki, et al., 2022). The samples were irradiated with a monochromatic CuKα radiation of 0.1542 nm between 2*θ* values of 5° and 90° at 0.05° intervals with a measurement time of 1 s per 2*θ* intervals. The films' thermograms were acquired on a Mettler Toledo TGA/DSC 30 (Mettler‐Toledo GmbH, Switzerland) (Madivoli, Kareru, Gachanja, et al., 2022; Madivoli, Kareru, Gichuki, et al., 2022). Approximately, 5 mg samples were weighed into 40‐μL aluminum crucibles that were then heated from 25 to 500°C and cooled to 25°C. Morphological analysis of the films was observed by acquiring scanning electron microscopy (SEM) micrographs of the films using Tescan Mira3 LM FE Scanning Electron Microscope (Tescan, Brno – Kohoutovice, Czech Republic) operating under an accelerating voltage of 3 kV. The samples were first coated with 4 nm gold using AGB 7340 Agar Sputter Coater before analysis (Agar Scientific, Essex, UK) (Madivoli, Kareru, Gachanja, et al., 2022; Madivoli, Kareru, Gichuki, et al., 2022).

### 2.8 Antimicrobial assay of composite films

The antibacterial activity was carried out using the disk diffusion agar method using Mueller–Hinton agar (MHA) media. This antibacterial activity was evaluated using the American type cell culture (ATCC), both Gram‐positive *Staphylococcus aureus* ATCC 25923, *Staphylococcus epidermidis* ATCC 12228, *Bacillus subtilis* ATCC 6051, and Gram‐negative *Escherichia coli* ATCC 25922, and *Pseudomonas aeruginosa* ATCC 27855 bacterial strains. The antibacterial activity was carried out following the Clinical and Laboratory Standards Institute (CLSI) guidelines (Madivoli et al., 2018; Odongo et al., 2023).

The (MHA) media was prepared as per the manufacturer's instructions and sterilized for 15 min using an autoclave at 121°C. The media was allowed to cool to about 50°C and then transferred into sterile disposable petri dishes and allowed to solidify. The inocula were prepared at 0.5 McFarland equivalent turbidity and adjusted to give a turbidity of 1 × 10^6^ cells or spores/mL (Odongo et al., 2023). The bacteria were spread using a sterile cotton wool swab, ensuring the microbes covered all the area of the plate (Hassan et al., 2019). The 6‐mm disks prepared from the composite film were placed on the inoculated plate followed by the addition of 5 μL solution of 1% dimethyl sulfoxide (DMSO) on top of the disk. The samples were then given 30 min for diffusion to take place and later incubated for 24 h at 37°C in an incubator. After the incubation period, the zones of inhibition were measured in millimeters (mm) using a vernier caliper as an indicator of the antibacterial activity of the films (Singh & Katoch, 2020).

## 3 RESULTS AND DISCUSSIONS

### 3.1 Optical properties

**FIGURE 3:**
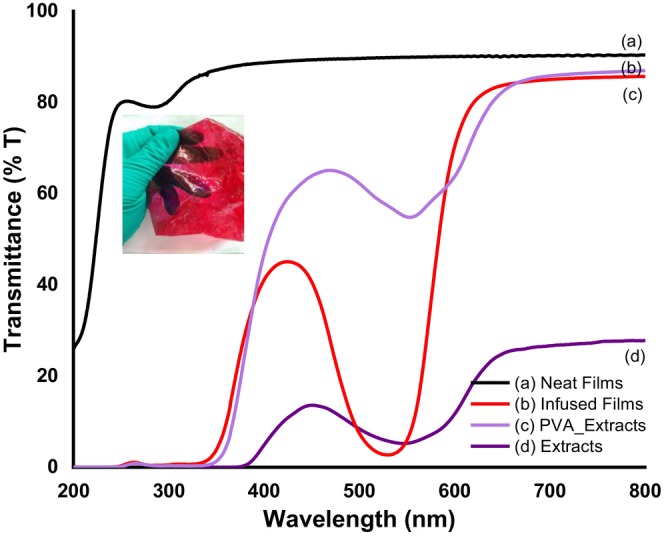
Ultraviolet (UV) spectra of (a) neat PVA–LA films, (b) PVA–LA films, and (c) PVA films infused with anthocyanins from *Brassica oleracea* var. *capitata* (d). Inset: Composite films infused with *B. oleracea* var. *capitata* extracts.

**FIGURE 4:**
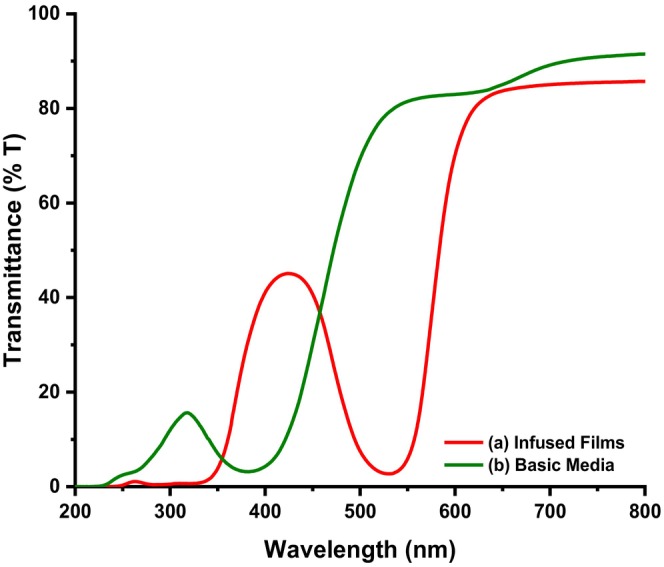
Ultraviolet (UV) spectra of composite films (a) acid pH and (b) basic pH.

The optical transparency of neat films and films infused with methanolic extracts of *B. oleracea* var. *capitata* and their corresponding response to basic pH are depicted in Figures 3 and 4.

From Figure 3a, neat composite films were observed to transmit ultraviolet (UV) radiation over the entire ultraviolet (UV) range, as compared to films infused with methanolic extracts from *B. oleracea* var. *capitata* (Figure 3b,c). In this case, it was observed that these films had an ultraviolet (UV) cut‐off at 550 nm, though a peak associated with anthocyanins (Figure 3d) was observed centered at 400 nm. Figure 4 shows that in the wavelength range of 280–315 nm, corresponding to ultraviolet‐B (UV‐B) radiation, the studied samples completely blocked the ultraviolet (UV) radiation and they had a very low transmission coefficient. The intense red color of the composite films caused a decrease in the visible light transmission of the films in the ultraviolet‐B (UV‐B) and ultraviolet‐R (UV‐R) range, which implies that they are an excellent packaging material to protect food and enhance shelf life during storage. It has been reported that the presence of anthocyanins in methanolic extracts of *B. oleracea* var. *capitata* is responsible for the broad absorption peak that was observed at 522 nm (Khoo et al., 2017; Roy & Rhim, 2021). This peak normally shifts with pH changes and in our case due to the acidic nature of the polymer solution, the peak shifted to 420 nm, leading to a change in color of the extracts from purple–violet to violet–red color in the resultant cast film (Figure 3). Upon exposure of the films to basic pH, the hypsochromic shift observed (Figure 4) was attributed to changes in the anthocyanin's molecular structure as the pH of the films changes from acidic to basic and vice versa (Khoo et al., 2017; Roy & Rhim, 2021). This makes these pigments unique compared to other natural colorants and the most preferred when designing smart packaging materials. In acidic conditions, some of the anthocyanins appear red, in neutral pH they have a purple hue while increasing the pH the color changes to green, hence the observed color changes in the composite films (Khoo et al., 2017; Roy & Rhim, 2021). In addition, due to their ability to absorb ultraviolet (UV) radiation and the nontoxic nature, their incorporation within the polymeric matrix for development of smart food packaging materials does not pose a health risk as compared to other pH‐responsive moieties (Khoo et al., 2017; Roy & Rhim, 2021).

### 3.2 Moisture content, solubility, and water vapor barrier properties (water vapor transmission rate (WVTR))

**FIGURE 5:**
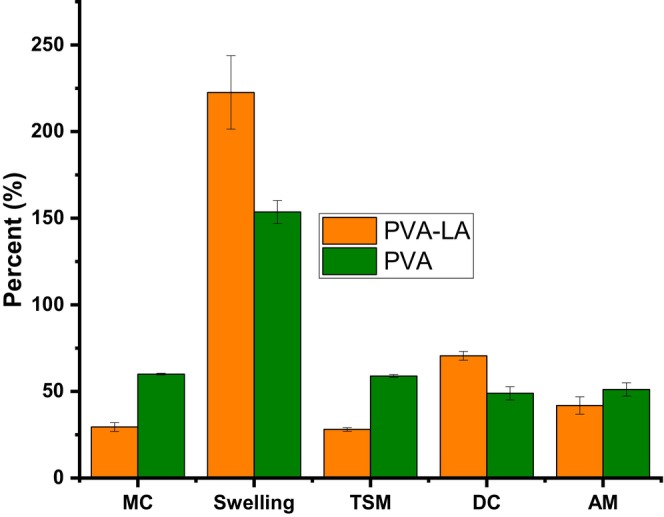
The moisture content (MC), degree of swelling (DS), total soluble matter (TSM), dry content (DC), and ATRO moisture content (AM) of the PVA–LA composite and PVA.

Figure 5 depicts the results of the physical properties of the films that were investigated in terms of moisture content, swelling, total soluble matter, dry content, and ATRO moisture content of the films.

From Figure 5, it was observed that incorporation of lactic acid into the PVA matrix, which resulted in an esterification reaction between hydroxyl group in PVA and carboxyl group in lactic acid, resulted in changes in the physical properties of the films. In this case, while this reaction resulted in a profound increase in the swelling degree and dry content, it resulted in a decrease in the moisture content, total soluble matter, and ATRO moisture content (Bhatia et al., 2023; Jayakumar et al., 2023; Otenda, Kareru, Madivoli, Salim, et al., 2022). The increase in the degree of swelling of the PVA–LA films is beneficial to the meat preserved in it, as it implies that the composite films could be able to absorb fluids emitted during storage before decay. It should be noted that the presence of moisture plays an important role in the growth of microorganism responsible for food decay, hence their absorption by the films hinders their proliferation. On the other hand, decrease in moisture content and the total soluble matter implies that the packaging will be able to absorb more fluids and the films will not disintegrate and dissolve during storage leading to food contamination by the film matrices (Bhatia et al., 2023; Jayakumar et al., 2023; Otenda, Kareru, Madivoli, Salim, et al., 2022). On the other hand, the water vapor permeability (WVP) was calculated to be (14.32 ± 1.14) × 10^−3^ g^−1^ s^−1^ Pa^−1^. Water vapor permeability (WVP) is an important parameter in food packaging as it affects the quality and stability of packaged products. WVP should be maintained as low as possible, to decrease the dehydration process of food in order to keep it fresh and it is an essential property for packaging material that indicates water transfer between food and the surrounding.

### 3.3 Mechanical properties

**FIGURE 6:**
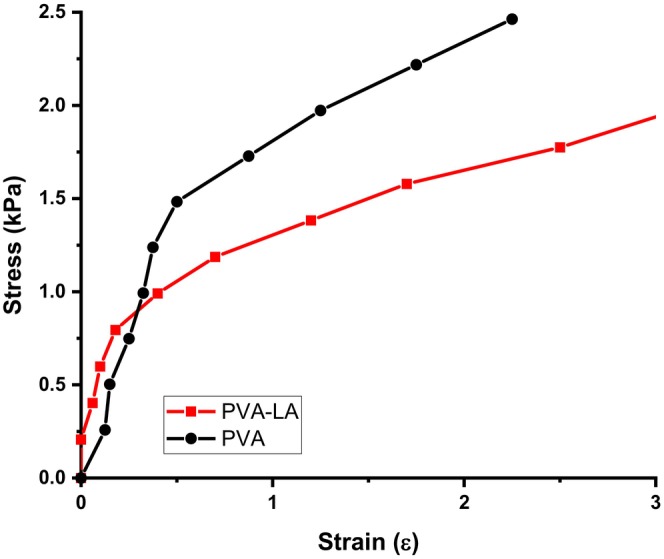
Stress–strain curve of PVA and PVA–LA composite films.

**FIGURE 7:**
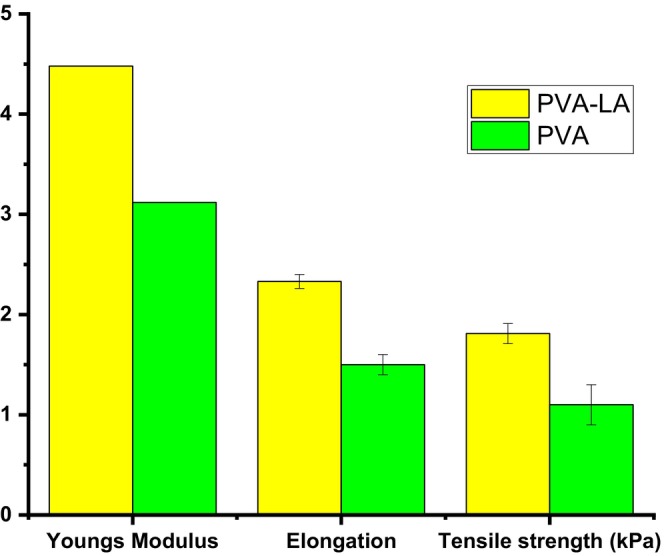
Young's modulus, elongation, and tensile strength of PVA–LA and PVA composite films.

**FIGURE 8:**
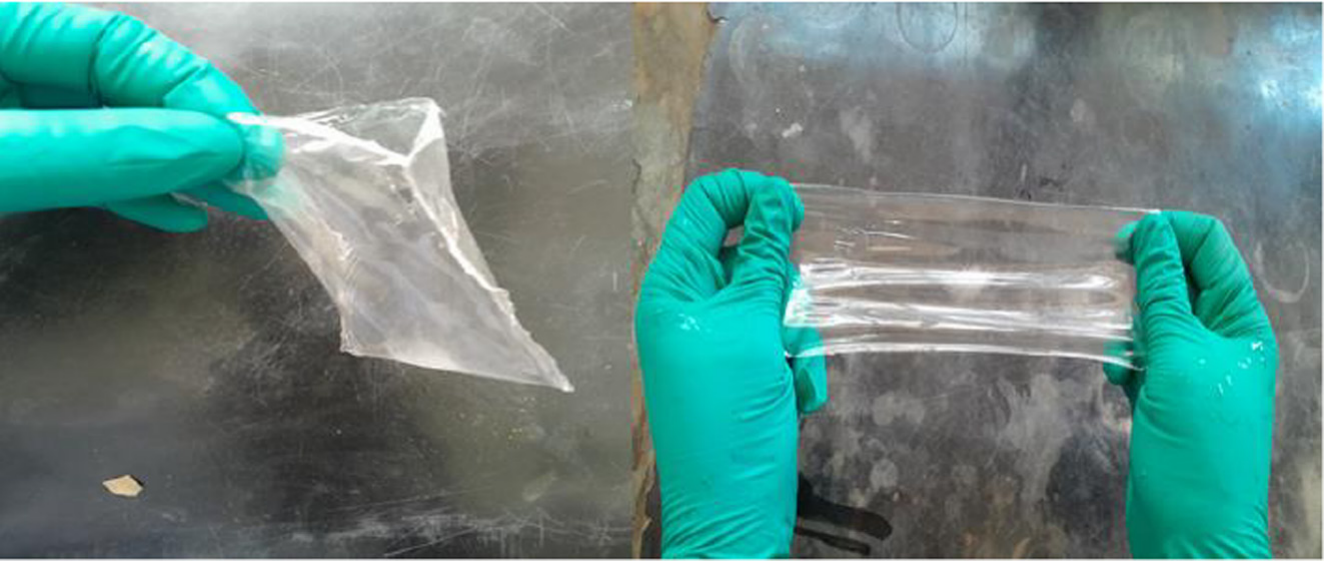
Neat films heat‐sealed to obtain a composite carrier bag.

Figures 6 and 7 depict the mechanical properties of the composite films, which are reported as stress, strain, Young's modulus, percent elongation, and tensile strength (Figure 8).

From Figure 6, it can be observed that neat PVA films fracture faster as compared to PVA–LA films, which implied that PVA films were more brittle while PVA–LA films were more flexible. On the other hand, it was observed that Young's modulus of PVA composite films increased upon incorporation of lactic acid within its matrix as compared to neat PVA films (Figure 7) (Bhatia et al., 2023; Jayakumar et al., 2023; Otenda, Kareru, Madivoli, Salim, et al., 2022; Wang et al., 2022). The addition of 50% lactic acid increased Young's modulus from 3.142 to 4.48 kPa as compared to pure PVA films (Figure 7). This indicated that lactic acid had strong bonding with PVA matrix through the esterification reaction that occurred during preparation. The most notable result is the significant improvement in the PVA films' elongation at break. PVA–LA films showed improvement in elongation at break, as the films achieved the highest elongation at break (116.3%) when compared to neat PVA films. This result indicated that incorporation of lactic acid with the PVA composite not only produced a high mechanical strength film but also remarkably enhanced the tensile strength, elongation at break, flexibility, and thermostability of the PVA–LA films (Bhatia et al., 2023; Jayakumar et al., 2023; Otenda, Kareru, Madivoli, Salim, et al., 2022; Sedlařík et al., 2006; Wang et al., 2022). From Figure 7, the enhancement of the elongation at break for the PVA–LA films may be due to the formation of strong interaction between the carboxylic groups of LA and the hydroxyl groups of PVA, leading to the formation of ester linkages. In a similar study, composite films prepared by 1:1 ratio of PVA–LA solutions exhibited the lowest strain at break when compared to PVA–LA solutions with the ratios of 1:2, 1:3, 1:4, and 1:5 but this was still much higher than for pure PVA. However, they reported that LA worked as a plasticizer as it increased the flexibility, workability, and extensibility of the rigid plastic, which is further aided by the good compatibility between the components due to their polarity, and changes in the free volume (Sedlařík et al., 2006).

### 3.4 Functional group analysis

**FIGURE 9:**
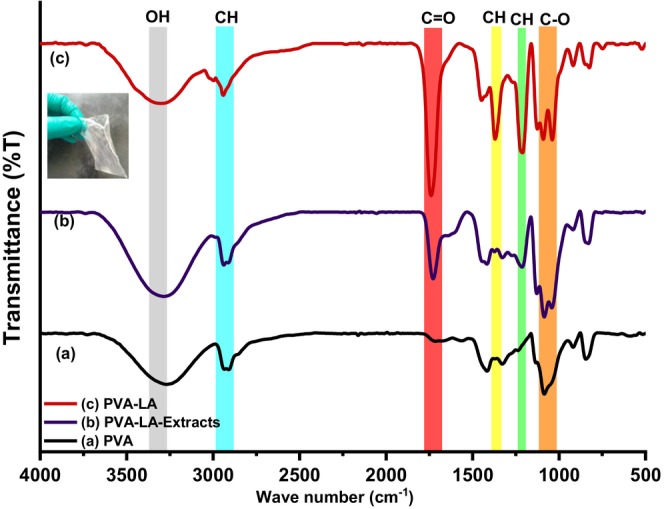
Infrared (IR) spectra of (a) PVA, (b) PVA–LA‐extracts, and (c) PVA–LA composite. Inset: Heat sealable films.

To better understand changes occurring during film formation and the esterification reaction between PVA and lactic acid, the functional groups formed were studied using infrared (IR) spectroscopy and the results are depicted in Figure 9.

From Figure 9, the IR spectra of PVA, PVA‐LA extracts, and PVA‐LA composite, it was observed that the composite films displayed vibrational bands typical of carboxylic functional groups, alcohols, and carbonyl, among others. The IR spectra of the neat PVA, PVA/LA (50:50) composite, and PVA–LA films loaded with anthocyanin extract films are shown in Figure 9. The peak at 3310 cm^−1^ was ascribed to the OH stretching of PVA, and it displays a significant shift from 3310 to 3345 cm^−1^. This results from the disruption of the O–H bonds in which OH groups are cleaved off to produce the ester linkage, typical of esterification reactions. This further evidenced to be a strong, sharp, and intense peak at 1757 cm^−1^, typical of C=O vibrational bands of esters that are formed during esterification reactions. With the exception of the significant shift relative to the OH stretching of PVA, the other peaks keep almost unshifted (Madivoli, Kareru, Gachanja, et al., 2022; Otenda, Kareru, Madivoli, Salim, et al., 2022; Wang et al., 2022). From Figure S1, upon polymerization of lactic acid to polylactic acid, the broad O–H stretching disappeared due to the formation of an acid dimer and subsequent polymerization to polylactic acid. From Figure 9a, the broad O–H stretching of carboxylic acids was centered at 3250 cm^−1^, whereas the C=O vibrational band was centered at 1750 cm^−1^ for lactic acid dimer (Madivoli, Kareru, Gachanja, et al., 2022; Otenda, Kareru, Madivoli, Salim, et al., 2022; Wang et al., 2022). Typically, carboxylic acid functional groups are characterized by a broad and intense O–H stretching vibrational band because of hydrogen bonding from 3500 to 2500, a carbonyl vibration at 1700 cm^−1^, and a C–O stretching vibration at 1250 cm^−1^. Similar observations have been reported in the literature where the esterification reaction between PVA and LA was observed from the C=O vibrational bands at 1204 and 1712 cm^−1^(Carlotti et al., 2001; Sedlařík et al., 2006). Similarly, these results reveal that only the OH group in LA is involved in this reaction and it corresponds to a work by Carlotti et al. (2001), where the partial formation of the ester was proved by nuclear magnetic resonance (NMR) measurements.

### 3.5 Thermal profile of composite

**FIGURE 10:**
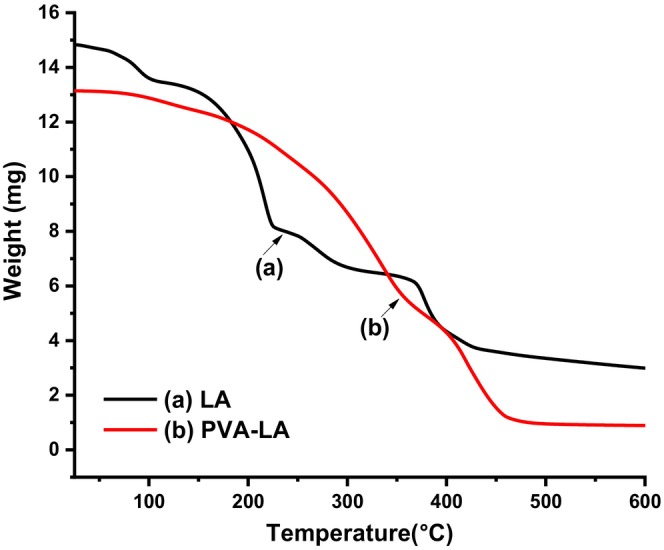
Thermal gravimetric analysis (TGA) thermograms of (a) LA and (b) PVA–LA composite films.

**FIGURE 11:**
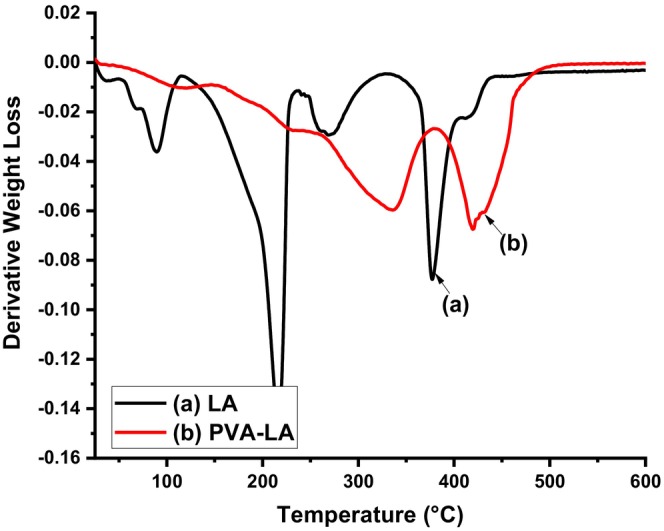
Derivative thermograms of (a) LA and (b) PVA–LA composite.

To better understand the thermal stability of the composite films, their thermal degradations were studied using TGA and the thermograms are depicted in Figures 10 and 11.

From Figure 10a, LA thermograms were characterized by three degradation cycles that were associated with evaporation of water at 95°C and the decomposition of the lactide at 390°C. At low temperatures, the thermal degradation of PLA is associated with the backbiting reaction from hydroxyl chain ends, while at higher temperatures, the thermal degradation is mainly caused by breaking of ester bonds, leading to cleavage of the PLA backbone (Chrysafi et al., 2021). On the other hand, LA had three degradation stages at 95, 220, and 390°C associated with water evaporation, decomposition of the acid dimer formed at higher temperatures, and breakage of anhydride bonds, respectively. In a similar study, TGA thermograms of composite films prepared from PVA–LA with 1:4 ratio were observed to have a maximum weight loss occurring at 230°C ascribed to LA desorption and volatilization. The results of TGA–IR also revealed that the three thermal decomposition stages observed in the thermogram were associated with vibration bands of PVA degradation and ester linkage. In this case, the volatile gases emitted during the thermal decomposition exhibited vibration bands at 2734, 1716, and 1177 cm^−1^ that are normally associated with LA esters. However, the vibrational bands of vinyl acetate that are observed at 1647 and 1769 cm^−1^ were absent in the TGA–FTIR spectra of PVA–LA due to the reesterification of residual PVA acetate groups by LA during film preparation (Carlotti et al., 2001; Sedlařík et al., 2006). It has been reported that the glass transition temperature (*T*_g_) and melting point temperature (*T*_m_) of PVA occur between 85–88°C and 200–230°C, respectively, and the degradation occurs at around 230°C (Carlotti et al., 2001; Sedlařík et al., 2006). Moreover, when compared to neat PVA films, PVA–LA films have been reported to have higher *T*_g_, *T*_m_, and *T*_d_ (thermal decomposition temperature) values due to the increasing hydrocarbon chain as a result of appreciable grafting that increases the intramolecular interactions.

### 3.6 X‐ray powder diffractograms

**FIGURE 12:**
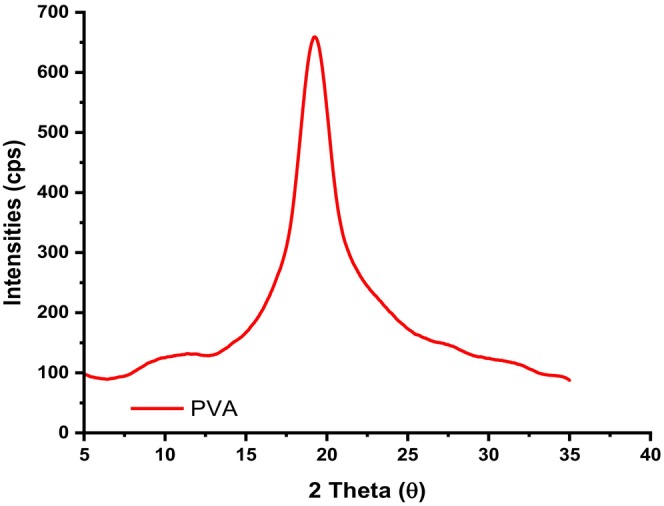
Powder diffractogram of PVA composite films.

**FIGURE 13:**
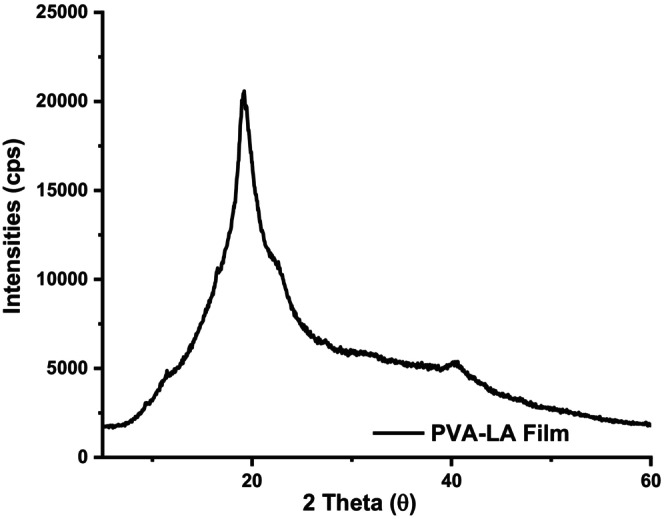
Powder diffractogram of PVA–LA composite films.

Figures 12 and 13 depict the powder diffractograms of neat PVA films and PVA–LA composite films after esterification reaction.

The main peak in the patterns at 2*θ* = 19° is the result of the superposition of the signals from the PVA crystallographic plane (101). When lactic acid is incorporated into the films, the degree of crystallinity of the resulting PVA–LA composite film does not change, as it retains the amorphous character which is characterized by a broad peak centered at 2*θ* = 19° (Figure 13). These results are in agreement with a study done by Sedlařík et al., 2006 where PLA–LA composite films exhibited a broad peak that was centered at 19.5^0^ associated with the presence of strong interconnected hydrogen bonds and assigned to the (1 0 1) diffraction plane (JCPDS File No. 41‐1049). Moreover, incorporation of two‐dimensional (2D) materials into host matrix showed X‐ray diffraction (XRD) patterns similar to that of PVA/LA, which was due to moderately low content of 2D filler in the polymer matrix (Acevedo et al., 2012; Carlotti et al., 2001; Monton et al., 2021; Sedlařík et al., 2006).

### 3.7 Film morphology and microstructure

**FIGURE 14:**
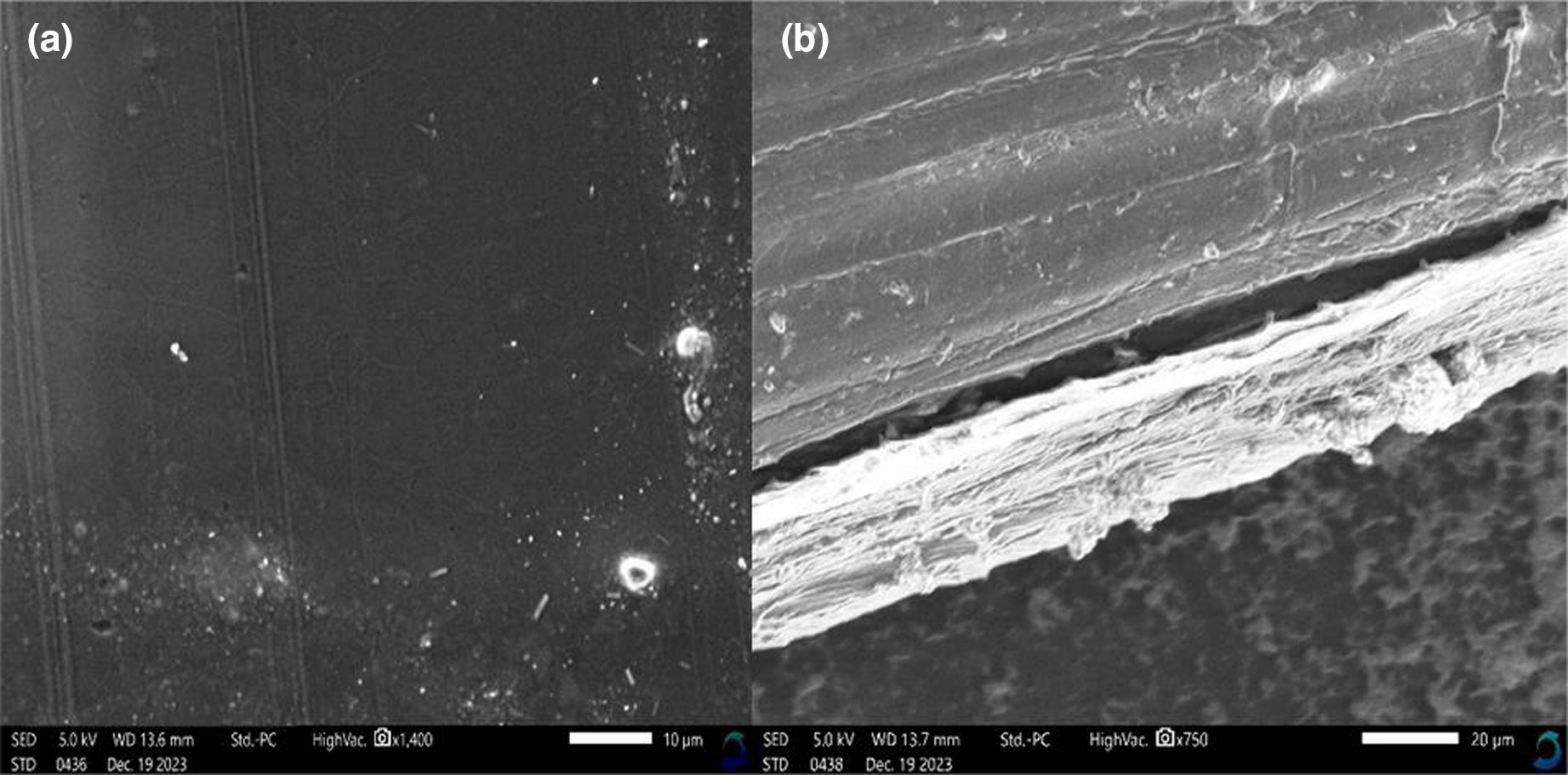
Scanning electron microscopy (SEM) micrographs of (a) film surface and (b) cross‐sectional area of films.

To better understand the morphological changes that occurred during films' preparation and encapsulation of anthocyanins, film microstructure was studied using SEM and the results are depicted in Figure 14.

From Figure 14, the composite films had a smooth surface with a few troughs here and there that can be attributed to the casting process and the smoothness of the cast. On the other hand, in some places, even rectangular sheets a few millimeters in length were visible, which was attributed to the presence of the anthocyanin extracts as these sheets were not present in the neat films (Figure S1). Moreover, the films were compact, dense, and had a greater strength interaction from esterification reaction between PVA and LA, leading to a reduction in the number of hydroxyl groups and the introduction of hydrophobic lactate (Acevedo et al., 2012; Carlotti et al., 2001; Monton et al., 2021; Sedlařík et al., 2006). However, it is interesting to note that the sheets were not evenly distributed throughout the composite films as they tended to be localized in composite films (Figure 14b). This localization of the sheets was attributed to the casting process and the lack of interaction between the extracts and polymer solution. This lack of interaction implies that the anthocyanins can easily diffuse from the films if appropriate conditions such as availability of a solvent can necessitate their diffusion from the films (Acevedo et al., 2012; Carlotti et al., 2001; Monton et al., 2021; Sedlařík et al., 2006). In this case, due to their reported antimicrobial and antioxidant activity, it implies that their encapsulation in food packaging materials will ensure that the stored food is not easily contaminated by microorganism and the onset of proliferation can actually be delayed, thereby increasing shelf life (Acevedo et al., 2012; Carlotti et al., 2001; Monton et al., 2021; Sedlařík et al., 2006). In a similar study, PVA–LA films loaded with propranolol hydrochloride exhibited a slow but continuous in vitro drug release of films prepared for transdermal drug release. The in vitro release of propranolol hydrochloride from PVA‐g‐LA film was found to be 2.60 ± 0.34 mg cm^−2^ (61.94 ± 8.03%) after 24 h. The patterns of in vitro release of propranolol hydrochloride from the film were low due to strong interactions between the drug and PVA–*g*–LA (Acevedo et al., 2012; Carlotti et al., 2001; Monton et al., 2021; Sedlařík et al., 2006).

### 3.8 Compound identification using GC–MS

**FIGURE 15:**
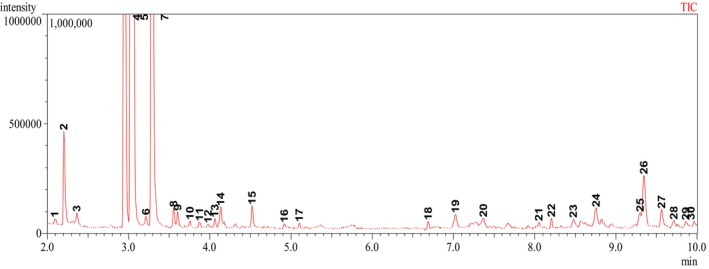
Gas chromatography–mass spectrometry (GC–MS) chromatogram of *Brassica oleracea* extracts.

Figure 15 and Table 1 depict GC–MS chromatogram and the list of compounds identified in the composite films infused with *B. oleracea* extracts.

**TABLE 1:** Compound identified in the hexane fractions.

Retention time	Retention indices	Compound name
2.092	27,395	3‐Pentanol, 3‐methyl‐
5.103	25,028	Undecane, 3,7‐dimethyl‐
7.023	128,303	Tetradecane
7.366	86,580	Pentadecane
8.052	29,666	Tetradecane, 5‐methyl‐
8.205	46,987	Nonane, 5‐(2‐methylpropyl)‐
8.756	136,604	2,6,10‐Trimethyltridecane
9.298	70,690	Tridecane, 6‐propyl‐
9.345	386,960	Eicosane
9.713	41,352	Octadecane, 5‐methyl‐
9.963	33,587	Tetradecane
11.631	73,051	Tetradecane
12.402	17,948	Hexadecane, 7,9‐dimethyl‐
12.544	40,328	Heptadecane, 2,6,10,15‐tetramethyl‐
13.423	19,592	Disulfide, di‐tert‐dodecyl
14.417	61,770	Tridecane, 5‐propyl‐
14.840	220,550	Neophytadiene
15.069	24,363	2‐Methyltetracosane
15.182	13,295	Hexadecane, 1‐iodo‐
15.234	33,713	Tetracosane
15.340	41,376	3,7,11,15‐Tetramethyl‐2‐hexadecen‐1‐ol
15.412	32,153	2,6,10‐Trimethyltridecane
15.497	20,381	Sulfurous acid, hexyl pentadecyl ester
15.915	80,582	Hexadecanoic acid, methyl ester
16.422	29,832	Phthalic acid, 4,4‐dimethylpent‐2‐yl butyl ester
16.611	33,876	Heneicosane
16.881	50,016	Isopropyl palmitate
17.726	33,601	9,12,15‐Octadecatrienoic acid, methyl ester (Z,Z,Z)‐
17.981	69,087	l‐Norvaline, N‐(2‐methoxyethoxycarbonyl)‐, hexadecyl ester
19.510	168,878	2‐Methylpentacosane
21.017	129,131	5‐Butyl‐5‐ethylheptadecane
21.186	49,143	5,5‐Diethylheptadecane
22.837	35,765	Heptacosane
23.125	15,512	Octacosane, 1‐iodo‐
23.530	911,340	gamma‐Sitosterol
24.011	23,660	Pentadecane, 8‐hexyl‐
24.291	73,684	Squalene
25.450	1,361,356	Octadecane, 1‐chloro‐
25.761	423,686	perhydro‐1,2,4a,6b,9,9,12a‐heptamethyl‐10‐hydroxy‐6a,14a‐Methanopicene
27.671	3,207,463	alpha‐Amyrin
29.346	225,958	Octacosan‐14‐one

From Table 1, it was observed that the hexane fraction was mostly composed of hydrocarbons that were fractionated from the methanolic extracts of *B. oleracea* extracts. Other compounds that were identified in the hexane fraction include terpenoids, amino acids, sesquiterpenes, ketones, esters, and carboxylic acids, such as norvaline, octacosan‐14‐one, hexadecanoic acid, 2‐hydroxy‐1‐(hydroxymethyl)ethyl ester, heptacosane, 1‐iodo‐octacosane, gamma‐sitosterol, 8‐hexyl‐pentadecane, squalene, and 1‐chloro‐octadecane, among others. The composition of the hexane extracts, which included straight chain alkanes, fatty acids, methyl esters, and aromatics, has been reported to have antioxidant and antimicrobial properties (Ashraf et al., 2018; Madivoli et al., 2018; Mwitari et al., 2013). For instance, extracts from *Daphne mucronata* leaves and stem were reported to contain pentadecane, 2‐methyl hexadecane, 7,9‐dimethyl hexadecane, tetradecane, 5‐propyl decane, 2,3,5,8 tetramethyl hexadecane, 2‐methyl‐6‐propyl dodecane, and 5‐methyl tetradecane, which were linked to the antioxidant and antimicrobial activity of the plant (Ashraf et al., 2018). Similarly, heneicosane, which is also present in the hexane fraction, has been shown to exhibit antimicrobial activity against *Streptococcus pneumoniae* 31 ± 0.64 mm and *Aspergillus fumigatus* 29 ± 0.86 mm, respectively, at 10 μg mL^−1^ concentrations (Vanitha et al., 2020). On the other hand, alpha‐amyrin has been shown to exhibit protein kinase A inhibition, immunostimulation, anti‐inflammatory, and antiplasmodic properties, while 2‐methyl‐3‐pentanol and neophytadiene have been shown to possess antimicrobial properties. As such, when the crude extracts are incorporated/infused into the polymer matrix, they enhance the antimicrobial and antioxidant activity of the resultant composite material, thereby increasing food shelf life by inhibiting the growth of food pathogens or food spoiling microorganisms (Otenda, Kareru, Madivoli, Salim, et al., 2022; Oulahal & Degraeve, 2022; Severo et al., 2021).

### 3.9 Detection of biogenic amines

**FIGURE 16:**
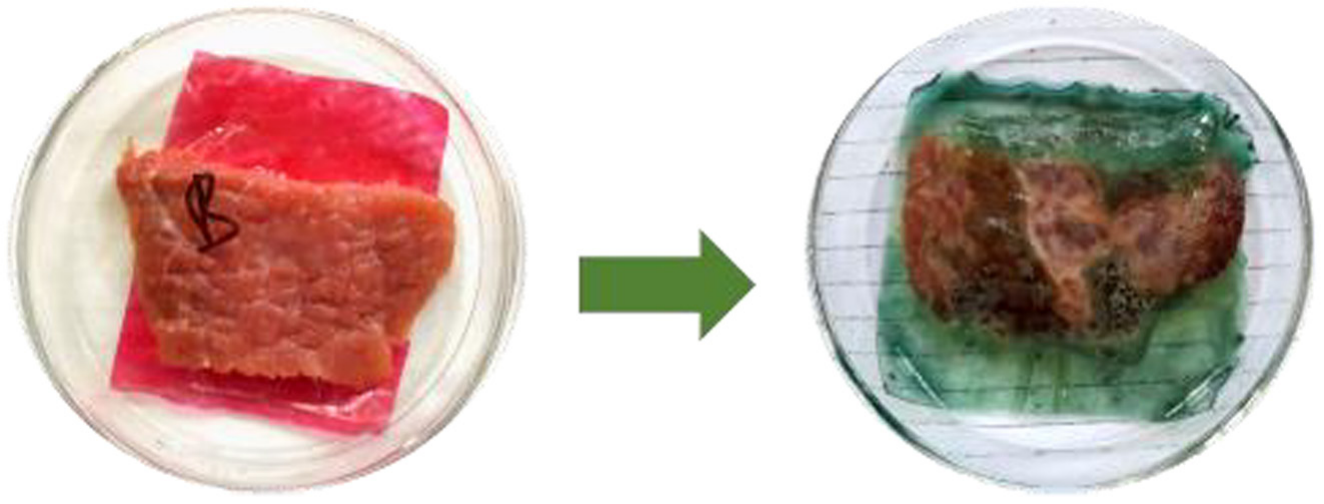
Changes in color of composite films before red and after 5 days in green.

**FIGURE 17:**
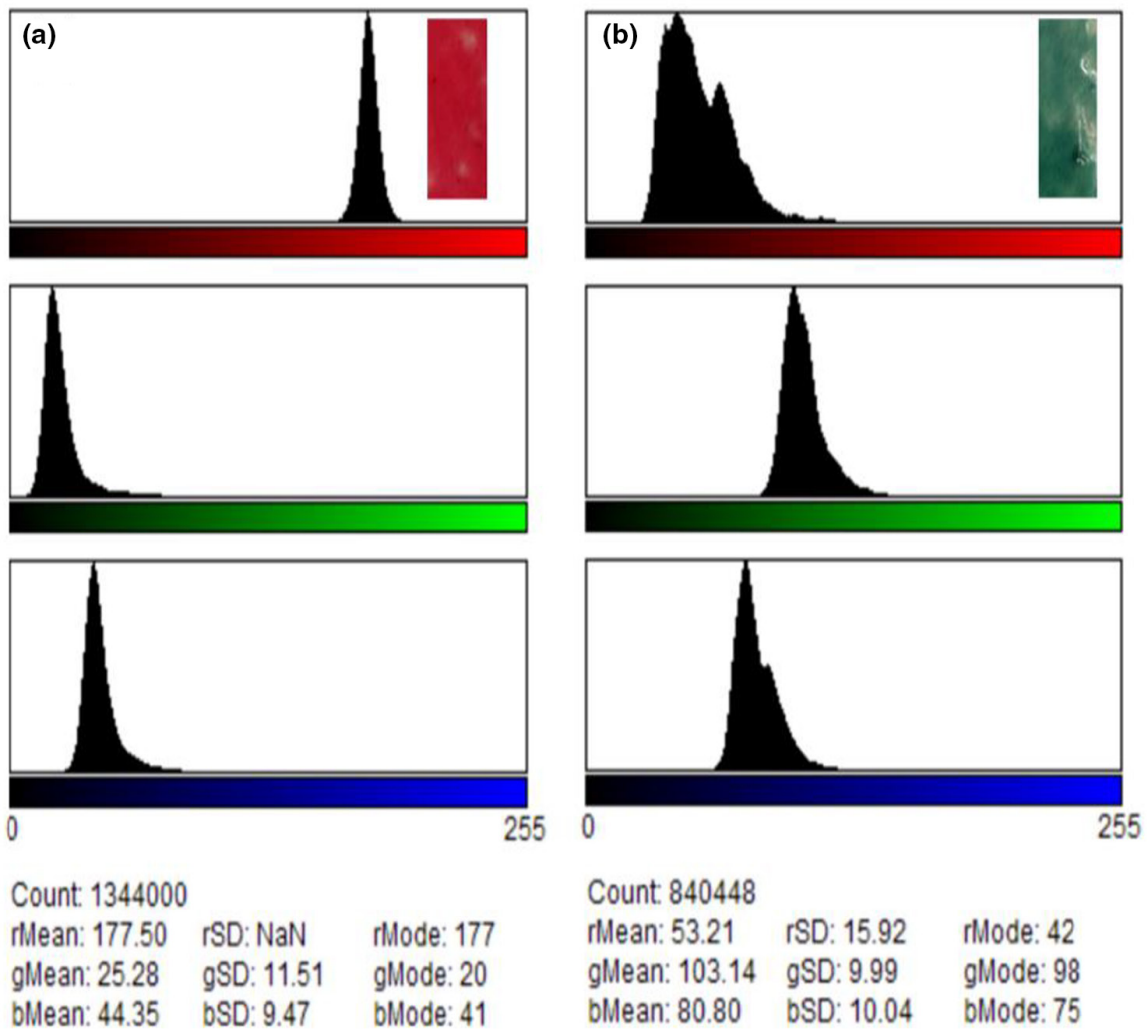
Red–green–blue (RGB) color histograms of the films (a) before and (b) after exposure to biogenic amines.

Figures 16 and 17 depict the changes observed in the color of the films upon wrapping beef for 5 days and their corresponding ultraviolet (UV) spectra.

**FIGURE 18:**
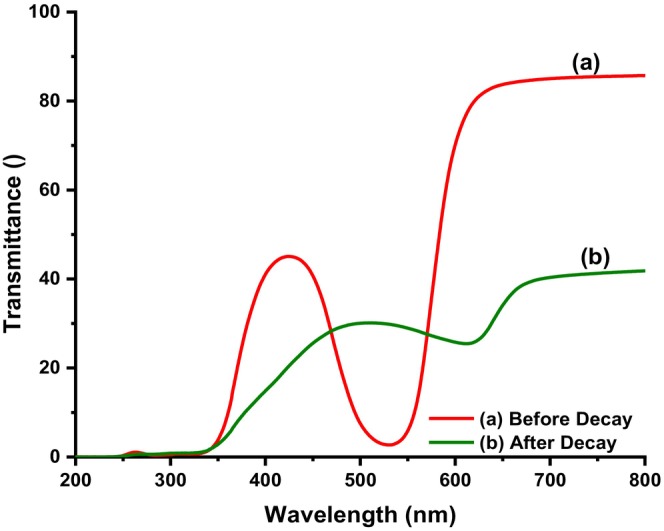
Ultraviolet (UV) spectra of composite films (a) before and (b) after meat decay.

As it can be observed from Figures 16, 17, 18, the color of the composite films changed from red to green after storing beef at ambient conditions for 5 days with a corresponding change in RGB values (Figure 17) and ultraviolet (UV) spectra (Figure 18) in which a bathochromic shift was observed (Acevedo et al., 2012; Carlotti et al., 2001; Monton et al., 2021; Sedlařík et al., 2006). In this case, the broad peak originally centered at 430 nm shifted to 500 nm as a result of contact between biogenic amines released from the meat during storage until decomposition (Figure 18) (Bhatia et al., 2023; Jayakumar et al., 2023). The colorimetric response of composite films at different pH values is depicted in Figure 18. It should be noted that the pH values correspond to the pH values of lactic acid, ammonia, methyl amine, dimethyl amine, and NaOH solutions, which were 4, 11, 12, 13, and 14, respectively (Miller et al., 2023; Weston et al., 2020). As it can be observed, the presence of lactic acid in the composite films lowered the pH of the resultant solution and when the extract was incorporated into the solution and cast, the resultant films were wine red. Exposure of these films to ammonia, methyl amine, dimethyl amine, and sodium hydroxide solution resulted in a change in the films' color from wine red to dark green, light green, and yellowish green colors for ammonia, methyl amine, dimethyl amine, and sodium hydroxide solutions, respectively (Miller et al., 2023; Weston et al., 2020). This resulted in changes in RCS values, as it can be observed from Figure 18. In a similar study, Weston et al., 2020 reported that exposure of anthocyanin–PVA films with an initial purple–pink color to lactic acid solution at pH 4 resulted in a change in color and RCS value of the resultant films from an RCS value of 27.0 ± 1.6% to 27.9 ± 1.7% attributed to the acidic nature of PVA films in solution (Miller et al., 2023; Weston et al., 2020). In this study, the changes in film RCS values were attributed to the interaction of PVA–LA films with organic chemicals, such as ammonia, hydrogen sulfide, and dimethyl amine, which are normally released during meat decomposition (Erkmen & Faruk Bozoglu, 2016; Miller et al., 2023).

### 3.10 Antimicrobial assays

**FIGURE 19:**
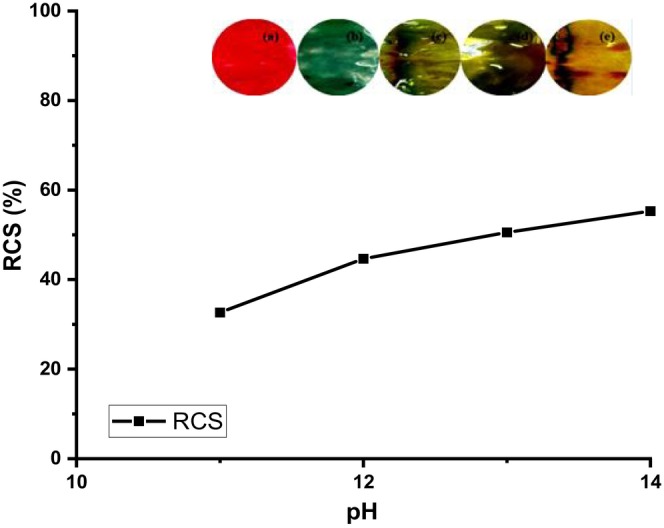
Red chromatic shift (RCS) values at different pH values. Inset: (a) Film before exposure, (b) in ammonia (pH 11), (c) in methyl amine (pH 12), (d) in dimethyl amine (pH 13), and (e) in sodium hydroxide (NaOH) (pH 13).

**FIGURE 20:**
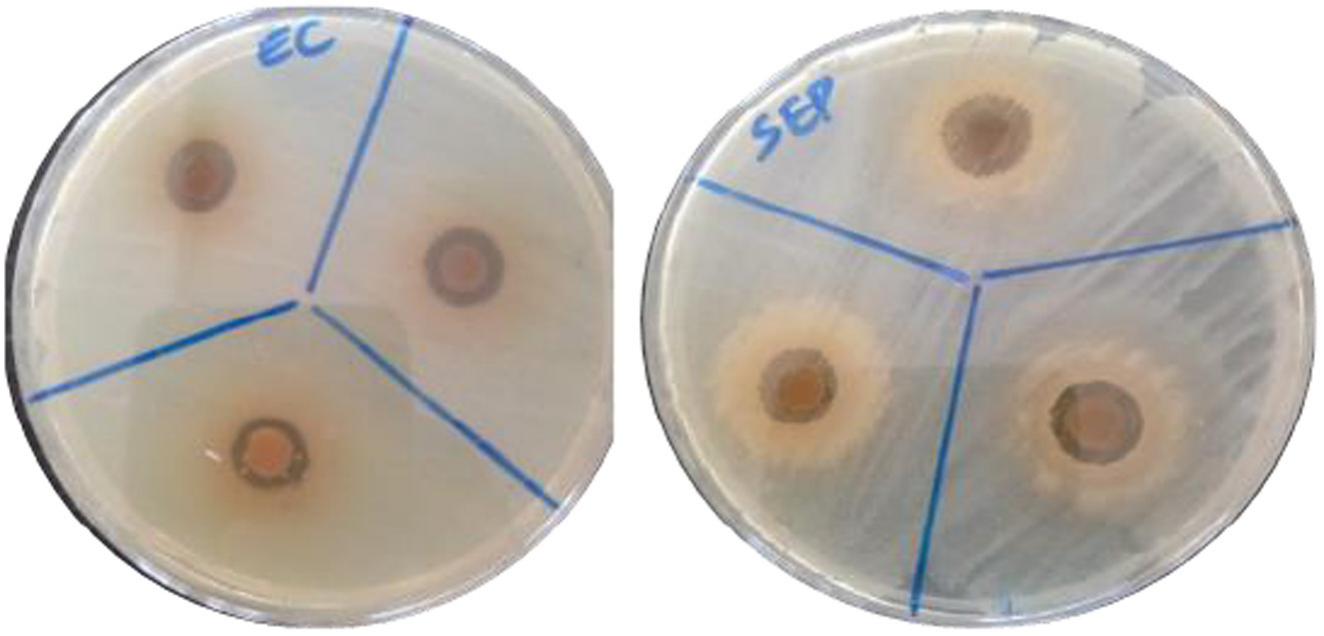
Antimicrobial assay of the composite films against selected foodborne pathogens.

**FIGURE 21:**
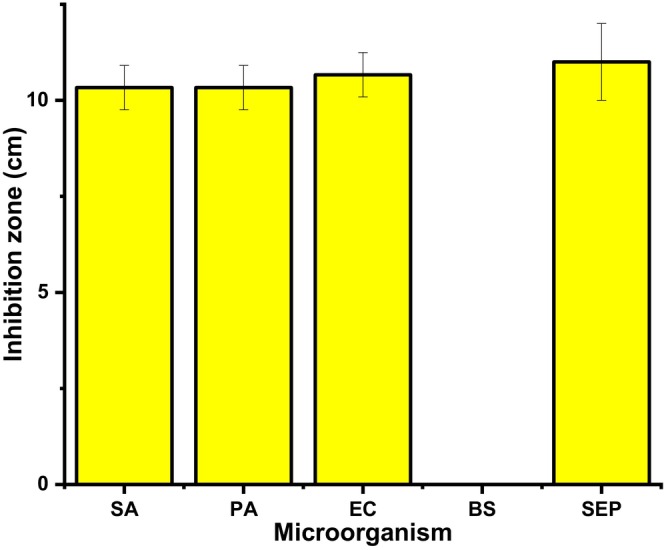
Antimicrobial assay of the composite films against *Staphylococcus aureus* (SA), *Pseudomonas aeruginosa* (PA), *Escherichia coli* (EC), *Bacillus subtilis* (BS), and *Staphylococcus epidermidis*.

The antimicrobial activity of the composite films infused with the methanolic extracts of *B. oleracea* was evaluated and the results are depicted in Figures 19, 20, 21.

Figures 19, 20, 21 depict the zone of inhibition profiles of the composite films evaluated against Gram‐positive; *S. aureus*, *B. subtilis*, and *S. epidermidis* and Gram‐negative; *E. coli* and *P. aeruginosa*. In the absence of methanolic extract, neat PVA–LA films did not inhibit the growth of the selected microorganism, hence they could not prevent food contamination when used as packaging material. However, incorporation of the extracts into the composite matrix increased the zones of inhibition in a dose‐dependent manner, as it was observed that films containing *B. oleracea* extract increased from 15% to 25%, as evidenced by the decrease in microbial growth around the films. The films exhibited activity against *E. coli* (10.67 ± 0.58), *S. aureus* (10.50 ± 0.40), *P. aeruginosa* (10.33 ± 0.58), and *P. aeruginosa* (11 ± 1) but no activity against *B. subtilis*. This microbial activity was attributed to the presence of secondary metabolites from the crude extracts of *B. oleracea*, which were encapsulated in film matrix. Different plant extracts such as Prunus africana have been reported to have antimicrobial activity against the selected microorganisms; hence, their incorporation into polymeric matrix is a step forward toward development of active food packaging materials (Otenda, Kareru, Madivoli, Salim, et al., 2022).

## 4 CONCLUSION

This study successfully prepared a composite film utilizing the esterification reaction between polyvinyl alcohol and lactic acid to prepare a pH‐responsive packaging material that changes color upon exposure to amines. The pH responsiveness of the films was achieved by loading methanolic extracts of *B. oleracea* which contained several classes of compounds that were identified from GC–MS studies including anthocyanins into the films, thereby the change in upon contact with methyl amine ammonia and dimethyl amine. When tested as a packaging material for meat samples, the color of the composite films changed from red to green as a result of interaction of the films with biogenic amines which were released during the decay. In this regard, the colorimetric response of the composite films containing anthocyanins can be used as an indicator to check the freshness of meat during storage, hence an excellent packaging material.

## FUNDING INFORMATION

The authors acknowledge the financial support of Partnership for Skills in Applied Sciences, Engineering and Technology ‐ Regional Scholarship and Innovation Fund (PASET‐RSIF) (Grant No. RSIF/RA/008) and the Kenya National Innovation Agency. The authors also acknowledge the financial support of the Swiss National Science Foundation given to Edwin Madivoli under grant number IZSEZ0_200290 to conduct a scientific visit at the Department of Chemistry, University of Fribourg under the guidance of Prof. Katharina Fromm, Fromm group.

## CONFLICT OF INTEREST STATEMENT

The authors declare no conflicts of interest.

## ETHICAL STATEMENT

No ethical approval was required for the preparation of this manuscript.

## Supporting information


Figures S1–S4

## Data Availability

All data gathered in the scope of the study are embedded within the manuscript and they can be made available upon reasonable request.
