# Supplementary material for: Antimicrobial and food barrier properties of polyvinyl alcohol–lactic acid food packaging films
Source: Food Sci Nutr. 2024 Jun 21;12(9):6563–77. doi: 10.1002/fsn3.4291 (PMC11561809; doi:10.1002/fsn3.4291)
Supplement: Supplementary file 1 — Figures S1–S4 [file FSN3-12-6563-s001.rtf]

Supplementary files


Figure S1: SEM Micrographs of composite films infused with methanolic extracts


Figure S2: GC-MS chromatogram of hexane extracts

Figure S3: Compounds eluted from GC-MS


Figure S4: Compounds eluted from GC-MS
